# Recent Advances in the Management of Acute Variceal Hemorrhage

**DOI:** 10.3390/jcm10173818

**Published:** 2021-08-25

**Authors:** Alberto Zanetto, Sarah Shalaby, Paolo Feltracco, Martina Gambato, Giacomo Germani, Francesco Paolo Russo, Patrizia Burra, Marco Senzolo

**Affiliations:** 1 Gastroenterology and Multivisceral Transplant Unit, Department of Surgery, Oncology, and Gastroenterology, Padova University Hospital, Via Giustiniani 2, 35128 Padova, Italy; alberto.zanetto@yahoo.it (A.Z.); sarahshalaby18@gmail.com (S.S.); martina.gambato@gmail.com (M.G.); germani.giacomo@gmail.com (G.G.); francescopaolo.russo@unipd.it (F.P.R.); burra@unipd.it (P.B.); 2 Anesthesiology and Intensive Care Unit, Department of Medicine, Padova University Hospital, 35128 Padova, Italy; paolo.feltracco@unipd.it

**Keywords:** cirrhosis, decompensation, bleeding, varices, survival, infection

## Abstract

Gastrointestinal bleeding is one of the most relevant causes of death in patients with cirrhosis and clinically significant portal hypertension, with gastroesophageal varices being the most frequent source of hemorrhage. Despite survival has improved thanks to the standardization on medical treatment aiming to decrease portal hypertension and prevent infections, mortality remains significant. In this review, our goal is to discuss the most recent advances in the management of *esophageal* variceal hemorrhage in cirrhosis with specific attention to the treatment algorithms involving the use of indirect measurement of portal pressure (HVPG) and transjugular intrahepatic portosystemic shunt (TIPS), which aim to further reduce mortality in high-risk patients after acute variceal hemorrhage and in the setting of secondary prophylaxis.

## 1. Introduction

Gastrointestinal (GI) bleeding is the second most frequent decompensating event in cirrhosis [1], with gastroesophageal varices representing the most frequent source of bleeding. Despite significant advances in the management of this complication, development of acute variceal hemorrhage (VH) is still associated with a six-week mortality risk of ~15–20% [2]. In patients who recover from VH, the risk of rebleeding is influenced by the treatment of underlying portal hypertension, with ~60% of untreated patients that will experience recurrent bleeding within on to two years, in contrast with only ~30% of those receiving therapies that lower portal pressure [3].

In this review, we discuss the management of patients with cirrhosis presenting with *esophageal* VH, including both treatment of the acute event (first section) and strategies to prevent recurrent hemorrhage (second section). The management of *gastric* variceal hemorrhage requires specific consideration and has been recently reviewed in depth elsewhere [4], therefore, it will not be included in the present review.

## 2. Control of Hemorrhage

The main goals of therapy in hospitalized patients with cirrhosis presenting with acute upper GI bleeding are (a) to control bleeding and (b) to prevent early rebleeding and death. Management can be divided into *general* measures, before the source of bleeding has been identified, and *specific* measures, once upper endoscopy has determined that hemorrhage is from esophageal varices.

### 2.1. General Measures

In combination with initial systemic stabilization (i.e., protection of circulatory and respiratory status) and start of intravenous proton pump inhibitors (PPIs), as in any patient hospitalized with upper GI bleeding, specific nuances in the management of patients with cirrhosis include a *restrictive* transfusion strategy and the use of prophylactic antibiotic therapy [5,6,7,8]. Additional measures include management of both coagulopathy and therapy with PPIs. For patients with alcohol-related liver disease, immediate and sustained cessation of alcohol consumption is particularly important to improve liver function and reduce risks of further bleeding, decompensation and mortality by reducing liver damage and portal pressure [9].

#### 2.1.1. Blood Transfusion Strategy

The main driver for development of esophageal VH is clinically significant portal hypertension [10]. In a way, the acute loss of intravascular volume due to bleeding reduces splanchnic pressure and may lead to self-limitation or self-interruption of active hemorrhage. By contrast, a sudden restitution of intravascular volume is associated with a rebound increase in portal pressure, which in turn may lead to failure to control bleeding and/or early rebleeding [11]. In a seminal randomized control trial (RCT), a “restrictive” transfusion strategy (hemoglobin threshold for transfusion of 7 g/dL with target range of 7–9 g/dL) was associated with a significantly higher probability of survival compared with a “liberal” strategy (hemoglobin threshold for transfusion of 9 g/dL with target range of 9–11 g/dL) [12]. Therefore, current guidelines recommend initiating transfusions in cirrhosis when hemoglobin levels decrease to <7 g/dL, with a target level of 7–9 g/dL [5,6,7,8].

Restitution of intravascular volume should be managed with large peripheral lines (16–18 gauges), and blood loss has to be replaced by red blood packed cells [8]. Replacement of fluids and electrolytes is important to prevent pre-renal acute kidney injury, which is common in cirrhosis with GI bleeding and is associated with increased mortality [13]. Nephrotoxic drugs such as nonsteroidal anti-inflammatory drugs, non-selective beta-blockers (NSBBs), and other hypotensive drugs may be suspended during the acute course of VH [6]. As occurrence of acute decompensation may be associated with instability in the feeble hemostatic balance of decompensated cirrhosis, the need for invasive procedures should be evaluated carefully on an individual basis. As discussed below, clotting factors may be replaced only to correct an eventual dilutional coagulopathy, whereas there is no indication to prophylactically correct a prolonged prothrombin time or a low platelet count [5,6,7,8].

#### 2.1.2. Antibiotic Prophylaxis

Bacterial infections are observed in up to 50% patients with cirrhosis hospitalized for GI bleeding, and are associated with strong risks of failure to control bleeding, early re-bleeding and mortality [14,15,16]. A recent meta-analysis including 12 studies comparing antibiotic prophylaxis vs. either placebo or no intervention demonstrated that the administration of prophylactic antibiotics was associated with reduced all-cause mortality (relative risk (RR): 0.79, 95% CI: 0.63–0.98), infection-driven mortality (RR: 0.43, 95% CI: 0.19–0.97), risk of bacterial infection (RR: 0.35, 95% CI: 0.26–0.47), rebleeding (RR: 0.53, 95% CI: 0.38–0.74), and length of stay (mean difference −1.9 days, 95% CI: −3.8–0.02) [17]. Therefore, a timely, short-term course of prophylactic antibiotics is an important step in the management of patients with cirrhosis and VH, and shall be instituted as early as possible upon admission, before upper endoscopy [5,6,7,8].

Whether severity of cirrhosis affects the importance of prophylaxis is unclear. In fact, while the role of prophylaxis is incontrovertible in patients with most advanced liver dysfunction (Child B and C), in those with less advanced liver disease conflicting data have been reported. In one retrospective analysis, Child A patients had lower risks of infection in the absence of prophylaxis (2%), and no difference in mortality was observed in treated vs. non treated patients [18]. The same study showed that the use of antibiotics was associated with a substantial reduction in mortality in Child C class [18]. However, prospective data are required to evaluate whether antibiotic prophylaxis can be avoided in Child A and current recommendation is to administer prophylaxis in all patients with cirrhosis presenting with VH, independent of child [5,6,7,8].

Intravenous ceftriaxone (1 g/24 h) for 7 days is the first choice in patients belonging to Child B and C classes, in those who were on quinolone prophylaxis, and in hospitals in which there is a high frequency of quinolone-resistant bacteria. Norfloxacin 400 mg twice daily may be used in the other patients. However, due to widespread quinolone resistance, ceftriaxone (a third-generation cephalosporin) has become the antibiotic of choice [5,6,7]. As approximately 30% of infection are from multidrug resistance antibiotics bacteria [19], evaluation of local resistance, if doable, may further improve definition of antibiotic regimen and should be considered [8]. Prophylactic antibiotics should be administered for a maximum of seven days, and their use should not be extended after discharge from the hospital [5,6,7]. In patients discharged before Day 7, transition to an oral antibiotic with the goal of completing seven days of treatment may be considered [8].

In a recent, nationwide study from Spain including 1656 patients with cirrhosis hospitalized for VH between 2013 and 2015, Martinez et al. investigated current epidemiology and trends of bacterial infections in these patients [20]. Interestingly, despite prophylaxis as currently recommended by international guidelines [5,6,7], 20% of patients developed bacterial infections, particularly respiratory tract infection. Development of infection was observed early (median time from admission 3 days) and was independently associated with Child C class (odds ratio (OR): 3.1; 95% CI: 1.4–6.7), Grade III–IV encephalopathy at admission (OR: 2.8; 95% CI: 1.8–4.4), orotracheal intubation for endoscopy (OR: 2.6; 95% CI: 1.8–3.8), and placement of nasogastric tube/balloon tamponade (OR: 1.7; 95% CI: 1.2–2.4 and 2.4; 95% CI: 1.2–4.9, respectively) [20]. Such procedures should, therefore, be minimized whenever possible, particularly in patients with additional risk factors, and active screening for respiratory infections shall be performed in case of early clinical deterioration. Whether patients at risk for respiratory infection would benefit from tailored regiments of antibiotic prophylaxis, particularly in settings with high risk of resistant strains bacteria, it remains to be evaluated in further studies.

#### 2.1.3. Additional Measures

##### No Need for Correction of Coagulopathy

Hospitalized patients with decompensated cirrhosis have severe coagulopathy [21,22,23]. However, a prolonged prothrombin time does not reflect an increased bleeding tendency in these patients [21,23], and correction of INR by fresh frozen plasma should not be performed [5,6,7,8]. Not surprisingly, administration of recombinant FVII, which can correct prolongation of INR, was not associated with additional benefit compared with standard of care in an individual patient data meta-analysis of two RCTs [24]. Administration of plasma to correct coagulopathy in cirrhosis with bleeding is a very common practice [25]; however, this practice not only is ineffective, but is also likely harmful [26]. In a recent, multicenter cohort study administration of fresh frozen plasma in cirrhosis with VH was independently associated with increased risks of 42-day mortality (primary outcome, OR: 9.41, 95% CI: 3.71–23.90), failure to control bleeding at five days (OR: 3.87, 95% CI: 1.28–11.70) and length of stay (adjusted OR: 1.88, 95% CI: 1.03–3.42) (secondary outcomes) [27]. No specific data exist regarding the management of severe thrombocytopenia in the setting of VH, and therefore, no recommendation can be made. In patients without chronic liver disease, desmopressin increases levels of plasmatic Von Willebrand factor/procoagulant Factor VIII, and its use was associated with reduced bleeding time in an old study including compensated patients [28]. However, in a subsequent RCT no difference in control of VH was observed between patients randomized to terlipressin alone vs. patients treated with terlipressin plus desmopressin [29]. Therefore, desmopressin is not currently recommended.

##### Limited Usefulness of PPIs

As peptic ulcers are the source of bleeding in ~30% of patients with cirrhosis presenting with GI bleeding [30], intravenous PPIs should be initiated as soon as possible. However, when portal hypertensive bleeding is confirmed at endoscopy, discontinuation of PPIs may be considered as they have shown no efficacy in this clinical setting. Limited evidence suggested that a short-term use (10 days) of PPIs might reduce banding ulcer size [31], however, this was not associated with a significant reduction of bleeding risk. PPIs in decompensated cirrhosis are associated with significantly increased risks of hepatic encephalopathy, bacterial infection, and readmission at 30-days [32,33,34]. In a landmark analysis including 1198 patients from three RCTs evaluating the use of satavaptan in patients with cirrhosis and ascites, Dam et al. demonstrated that the use of PPIs was associated with a significantly increased risk of encephalopathy (OR: 1.88, 95% CI: 1.21–1.91) and spontaneous bacterial peritonitis (OR: 1.72, 95% CI: 1.10–2.69) during follow-up [33]. Recent data with extended period of follow-up confirmed that regular use of PPIs not only is associated with increased risk of spontaneous bacterial peritonitis, but also predicts liver-related mortality independent of MELD and stage of cirrhosis (OR: 2.01, 95% CI: 1.38–2.93) [34]. Therefore, their use should not be extended past discharge.

### 2.2. Specific Management of Acute Esophageal VH

Standard therapy for acute VH includes intravenous splanchnic vasoconstrictors and placement of rubber bands around esophageal varices, especially the one that is expected to be the source of bleeding [5,6,7,8]. Endotracheal intubation to protect the airway system may be considered in patients with massive bleeding prior to endoscopy. However, whether intubation is really protective or increases the risk of respiratory infections is unclear [20], therefore, it cannot be recommended for every patient.

#### 2.2.1. Intravenous Splanchnic Vasoconstrictors

Three intravenous splanchnic vasoconstrictors are available: terlipressin, somatostatin or octreotide. These drugs exert their action by reducing splanchnic blood flow, therefore lowering portal pressure [35]. They are very effective and a recent meta-analysis clearly demonstrated that the use of vasoconstrictors is associated with a significantly higher probability of bleeding control and a lower seven-day mortality [36]. As a proof of concept, treatment with vasoconstrictors alone was previously found to control bleeding in >80% of patients [37]. It is most likely the widespread adoption of these drugs, together with the optimization of general medical care, that has significantly lowered the VH-related short-term mortality in the recent years [38].

A vasoconstrictor shall be initiated as soon as possible and early administration is associated with improved survival [5,6,7,8]. A placebo-controlled trial in which terlipressin was administered during the ambulance transfer showed that such early timing of administration was associated with increased probability to control of bleeding and survival in the treatment arm [39].

In clinical practice, the choice among these three intravenous vasoconstrictors is dictated by local availability and cost [40]. Recommended dose for terlipressin of 2 mg/4 h during the first 48 h, followed by 1 mg/4 h. If terlipressin is contraindicated, somatostatin is an alternative and should be administered as a continuous infusion of 250 mg/h (that can be increased up to 500 mg/h), with an initial bolus of 250 mg. The recommended dose of octreotide is a continuous infusion of 50 mg/h with an initial bolus of 50 mg [5,6,7,8].

Vasoconstrictors should be continued up to five days after the confirmation of VH because the risk of rebleeding during this time is particularly high [5,6,7,8]. However, as vasoconstrictors may be associated with potentially serious adverse events, the feasibility of a shorter administration (i.e., 24–48 h vs. 3–5 days) has been considered. In a recent meta-analysis, although the risk of 42-day mortality was not significantly different between one to three and five days, risk stratification was missing [41]. It may be that Child A patients could receive a shorter duration of therapy, whereas all others would require five days, but further studies are required to answer this question.

In summary, guidelines recommend that an intravenous splanchnic vasoconstrictor shall be initiated as soon as possible, prior to diagnostic endoscopy, and be administered for three to five days [5,6,7,8].

#### 2.2.2. Endoscopic Therapy

Once hemodynamic stability has been reached, an upper GI endoscopy shall be performed to determine the cause of bleeding and to provide specific treatment [42]. Early data and one relatively recent retrospective study suggested that endoscopy within 12 h from the index event might be associated with reduced rates of recurrent bleeding and mortality [43]. On the other hand, in a larger multicenter study including 1373 patients with cirrhosis and VH, endoscopy within 24 h from admission was associated with lower mortality in patients with Child A or B cirrhosis (OR: 0.38, 95% CI: 0.16–0.86; *p* = 0.020) and in those with systolic blood pressure <90 mmHg (OR: 0.053, 95% CI: 0.006–0.51; *p* = 0.011) [44]. In contrast, performance of endoscopy within either 6 or 12 h was not associated with a further reduction in mortality compared with endoscopy within 24 h. Interestingly, the association between endoscopy within 24 h and reduced mortality was seen in Child A and B patients, but not in the overall group including also Child C [44].

This notwithstanding, current guidelines recommend that once hemodynamic stability has been achieved, endoscopy should be performed as early as possible, and within 12 h since presentation [5,6,7,8]. When VH is confirmed, either by the presence of a bleeding varix, a clot, or a “white nipple” over the varix, or when varices are the only abnormality observed that would explain the hemorrhage, all esophageal varices should be ligated, particularly the one that is considered the source of hemorrhage. Endoscopic variceal ligation (EVL) should be performed within the same endoscopy session. EVL is more effective than sclerotherapy, is associated with fewer adverse effects, and does not lead to further increase in portal hypertension [45]. Therefore, sclerotherapy should be restricted to the rare cases in whom ligation is not technically feasible. Hemostatic powder applied endoscopically may be considered as a rescue therapy, however, few data exist and its applicability remains to be determined [46,47].

In patients with uncontrolled bleeding, guidelines recommend placement of balloon tamponade (Sengastaken-Blackemore or Minnesota tubes) [5,6,7,8]. However, tamponade carries a high risk of complications, particularly respiratory infection [20], and shall be considered only as a temporary (maximum 24 h) bridge to TIPS [5]. Recent data suggest that placement of a self-expandable esophageal metal stent (placed orally or endoscopically) may be associated with greater bleeding control and lower adverse events compared to balloon [48]. As these stents may remain in place for up to 7 days, this would allow more time to plan for a definitive treatment. Per the last Baveno consensus, if available, the application of stents may be considered as a preferred alternative compared with balloons [5].

#### 2.2.3. Transjugular Intrahepatic Portosystemic Shunt (TIPS)

##### Rescue TIPS (in Patients Who Fail Standard Therapy)

Despite combination therapy with prophylactic antibiotics, intravenous splanchnic vasoconstrictors, and EVL, 10–15% of patients will have either persistent bleeding or early rebleeding, which are associated with high risk of death [49]. Negative predictors for failure to control bleeding or early rebleeding include Child C class, portal vein thrombosis, severity of portal hypertension as defined by a hepatic venous pressure gradient (HVPG) >20 mmHg, and systolic blood pressure < 100 mmHg at admission [50,51].

In patients with mild-moderate rebleeding, a second session of endoscopy with ligation may be attempted. In patients with persistent or severe rebleeding (i.e., those with failure of endoscopic therapy), rescue TIPS is the therapy of choice [5,6,7,8]. In fact, by connecting the hypertensive portal venous system to the normotensive system of inferior vena cava, TIPS will quickly reduce portal pressure and resolve bleeding.

Given the lack of therapeutic alternatives, the only factor that would limit the use of rescue TIPS is futility. One issue to consider is patient’s eligibility for transplantation. Additional factors include number and severity of organ failures. However, development of acute on-chronic liver failure (ACLF) per se is not an absolute contraindication for placement of rescue TIPS. In fact, in a recent multicenter study including 174 patients with either acute decompensation or ACLF and uncontrolled variceal bleeding, the insertion of rescue TIPS was an independent predictor of survival at 42 days [52]. There are multiple studies that looked for predictors of futility in patients undergoing rescue TIPS. In a recent, large multicenter cohort including 164 patients who received rescue TIPS, those with arterial lactate ≤ 2.5 mmol/L and MELD score ≤ 15 had 6-week survival > 85%, whereas those with baseline lactate level ≥ 12 mmol/L and/or MELD score ≥ 30 had >90% risk of death [53]. A recent, large observational study of rescue TIPS showed that stay in intensive care unit prior to TIPS, MELD, and Child-Pugh score were independently associated with mortality at six weeks, and the authors commented on the futility of rescue TIPS in patients with Pugh score > 13 [54].

##### Preemptive TIPS (In Patients at High-Risk of Failing Standard Therapy)

Patients with acute VH who are more likely to fail, despite initial control of hemorrhage by standard therapy, are those belonging to Child C class or those with HVPG > 20 mmHg [50,55]. It was, therefore, postulated that the placement of a TIPS before failure of standard therapy (“pre-emptive TIPS”) could improve survival [56]. A first RCT in which 52 patients with HVPG > 20 mmHg was randomized to standard treatment vs. pre-emptive TIPS (uncovered) demonstrated significantly lower failure rates and all cause short-term mortality in the TIPS arm (12% vs. 50% and 17% vs. 38%, respectively) [57]. When covered TIPS became the standard-of-care, a second RCT confirmed an improved survival in patients randomized to TIPS vs. standard of care [58]. Both the one-year rate of failure to control bleeding/rebleeding and mortality were decreased by TIPS, the absolute reduction being 47% and 25%, respectively [58]. In this RCT, high-risk patients were defined as those with Child C cirrhosis and a Pugh score of 10–13, or those belonging to a Child B class with active bleeding at time of endoscopy.

Later on, however, large international observational cohorts confirmed the beneficial effect on survival of pre-emptive TIPS only in Child C patients (score 10–13), but not in those with Child B and active bleeding [59,60]. Therefore, the inclusion of Child B with active bleeding at endoscopy was questioned for potentially overrating the risk of mortality.

A recent RCT from China including 132 patients in Child B and C class and randomized 2:1 to pre-emptive TIPS vs. standard of care reported better transplant-free survival at six weeks and one year (OR: 0.50, 95% CI: 0·25–0·98; *p* = 0.04) and improved control of bleeding or rebleeding with early TIPS (OR: 0.26, 95% CI: 0.12–0.55; *p* < 0.0001) [61]. Importantly, the survival benefit was found in all subgroups, independent of either active bleeding or stage of cirrhosis, and no difference was found in the rate of hepatic encephalopathy. However, the reduction in mortality risk was relatively small (one-year survival 86% vs. 73% in pre-emptive TIPS vs. standard of care, respectively), which likely reflects the inclusion of patients at relatively lower risk of failure (57% of patients were Child B with no active bleeding and proportion of Child C was only 22%) [61]. It is also important to note that 75% of patients had HBV-related cirrhosis, which could have influenced the outcomes and may limit applicability of these results to Eastern countries. Furthermore, sclerotherapy was used in more than 5% of patients in standard of care group, which is not in line with current guidelines [5,6,7].

Another RCT from England in 58 patients with Child–Pugh score ≥ 8 showed no difference in survival (OR: 1.154, 95% CI: 0.3289–3.422; *p* = 0.79) and risk of rebleeding between pre-emptive TIPS and standard treatment, independent of severity of cirrhosis or active bleeding. However, the study was underpowered and only 23/29 patients (79%) underwent preemptive TIPS, with only 13/23 within 72 h (therefore, not deemed early by definition) [62]. Remarkably, the one-year transplant-free survival in the control arm was higher than that in the 2010 RCT by Garcia-Pagan (76% vs. 61%) [58]. This may be related to the significant improvements in overall management of VH in cirrhosis, which would question the extrapolation of results from the 2010 study by Garcia-Pagan to present times [58]. On the other hand, an alternative explanation could be that patients included by Dunne et al. were not at high-risk of failure to control bleeding, which would not have allowed to assess the true benefits of pre-emptive TIPS [62].

Opposite to Dunne’s findings, a large meta-analysis with individual data from 1327 patients included in seven studies, of whom 602 were Child B with active bleeding, found that placement of pre-emptive TIPS was associated with improved survival not only in the overall group (OR = 0.443, CI 95%: 0.323–0.607, *p* < 0.001), but also when Child B (OR = 0.524, CI 95%: 0.307–0.896, *p* = 0.018) and Child C (OR = 0.374, CI 95%: 0.253–0.553, *p* < 0.001) patients were analyzed separately [63]. This would support the use of pre-emptive TIPS in both Child C and Child B patients with active bleeding. However, results in Child B are less convincing/consistent compared with those obtained in Child C. Additional limitations are the inclusion or more observational studies than RCTs (four versus three), definition of high risk patients by only one specific criterion (therefore, not being able to assess if additional criteria might have better classified patients with VH at high risk), and the heterogeneity in both TIPS expertise and treatments in standard of care arms across different centers [63].

Therefore, a multicenter trial collecting large numbers of patients undergoing pre-emptive TIPS remains a research priority in this field. Importantly, such trial should assess not only which group(s) of patients are most likely to benefit from pre-emptive TIPS, but also whether there is a maximum threshold of severity of liver disease above which there is no improvement of survival.

While awaiting such trial, guidelines recommend to consider pre-emptive TIPS in patients with Child C cirrhosis (score 10–13) [5,6,7,8]. Patients with Child A cirrhosis and those with Child B cirrhosis without active bleeding should not be considered for pTIPS. Further data are required before to make a strong recommendation in patients with Child B and active bleeding at time of endoscopy.

As one major goal of pre-emptive TIPS is to prevent development of ACLF, one would think that pre-emptive TIPS should not be considered in patients who have already developed ACLF at time of admission/decision making. However, a recent study from a European collaborative group found that ACLF is an independent predictor of bleeding-related mortality, and that pre-emptive TIPS may improve outcomes in selected patients with ACLF [64]. Although prospective data are needed, these preliminary findings indicate that ACLF per se is not an absolute contraindication for pre-emptive TIPS, and instead eligibility should be a case-by-case decision according to number and severity of organ failures.

Despite RCTs and observational cohorts have demonstrated that pre-emptive TIPS is associated with a survival benefit, and the use of pre-emptive TIPS in these patients has been recommended since the Baveno V consensus (published 11 years ago) [65], a recent French survey revealed that only 7% of eligible patients finally received a pre-emptive TIPS [60]. Similarly, in another large observational cohort, a pre-emptive TIPS was placed in only 13% of high-risk patients [59]. This indicates a significant underutilization of pre-emptive TIPS in real-life practice, which is somewhat concerning considering its substantial effect on patient survival. Further efforts are required to lower the bar for a widespread adoption of pre-emptive TIPS in daily practice. These efforts include creation of dedicated networks through which selected patients may be referred early to tertiary care centers with specific expertise in invasive management of portal hypertension and its complications.

## 3. Prevention of Recurrent Hemorrhage

Per current guidelines, patients who had a TIPS placed after VH do not require further medical or endoscopic therapy for secondary prophylaxis, and should instead be referred for liver transplant evaluation in case they have additional complications of cirrhosis [5,6,7,8]. Patency of TIPS should be assessed at regular intervals by doppler ultrasound together with screening for hepatocellular carcinoma.

### 3.1. First Line Therapy

Combined therapy with NSBBs (propranolol or nadolol) plus EVL is the first line therapy in prevention of rebleeding [5,6,7,8]. This recommendation is based on multiple meta-analyses of RCTs performed to prevent rebleeding. One of these meta-analyses demonstrated that the added effect of NSBBs to EVL improved the efficacy of EVL alone and reduced mortality, whereas the added effect of EVL to NSBB was only associated with a non-significant decrease of rebleeding with no effect on survival [66]. A recent individual patient data meta-analysis evaluated data from three trials comparing NSBBs vs. combination therapy and from four trials analyzing EVL vs. combination therapy [67]. As these were individual data, the authors were able to perform risk stratification in Child A vs. Child B/C patients. Interestingly, in Child A (mostly compensated), combination therapy was associated with lower all-source rebleeding, without an effect on mortality. In Child B/C patients (mostly decompensated), combination therapy was associated with lower all-source rebleeding rates only in trials in which it was compared to EVL alone, indicating that NSBBs alone could be enough to prevent all-source rebleeding in these patients. Importantly, mortality was also lower in trials in which combination therapy was compared to EVL alone, suggesting that NSBBs not only are essential in preventing rebleeding, but also death [67].

These data, obtained from RCTs, are in contrast with a previous cohort study including patients with refractory ascites in which mortality was significantly higher in those receiving NSBBs [68]. However, study groups were different at baseline and patients were sicker in the NSBB group. Additionally, the determination of NSBBs was evaluated at diagnosis of refractory ascites with no information on their use thorough follow-up. Multiple trials in different groups of patients with decompensated cirrhosis have been conducted to confirm or refute these findings, and two meta-analyses have summarized these data both showing that the use of NSBBs is not associated with a higher mortality [69,70].

In studies that showed a detrimental effect of NSBBs [68,71], the arterial pressure in NSBBs users was lower than that in non-users, and a higher dose of propranolol was used, or a higher percentage of patients were treated with carvedilol. This indicates that patients in whom a negative inotropic effect or a vasodilatory effect from NSBBs/carvedilol were the ones that were negatively affected by beta-blockers [72]. In a way, this can be expected as this clinically-evident, likely dose-related deleterious hemodynamic effect of NSBBs would worsen the already vasodilated state of decompensated patients, leading to renal hypoperfusion, renal failure and death [73]. Indeed, in a propensity-matched analysis including only patients with refractory ascites, the use of propranolol was associated with an increased survival, except for the subgroup on a high dose (160 mg/day or more) [74].

Propranolol and nadolol should be used cautiously in patients with ascites and should be started at a lower dose than in patients without ascites, and the maximum dose should be capped also at a lower dose: propranolol should be capped to 160 mg/day (320 mg/day in patients without ascites) and nadolol to 80 mg/day (160 mg/day in patients without ascites) [7]. Importantly, the dose of NSBB should be reduced or drug should be discontinued in patients with refractory ascites who developed circulatory dysfunction defined by systolic blood pressure < 90 mm Hg, serum sodium < 130 meq/L, or acute kidney injury [7].

In summary, current guidelines recommend that first line therapy to prevent recurrent VH is the combination of NSBBs (propranolol or nadolol) plus EVL, independent of the presence or absence of ascites/refractory ascites or other complications of cirrhosis [5,6,7,8].

However, it is possible that refinements in risk stratification could lead to identification of “higher” risk patients in whom an aggressive approach, such as placement of TIPS, may be beneficial as first line therapy (i.e., before development of recurrent VH). This was recently evaluated by La Mura and Bosch in a retrospective study including 424 patients with cirrhosis candidates to secondary prophylaxis [75]. Inclusion criteria were diagnosis of cirrhosis, admission for VH within the previous seven days, baseline HVPG ≥ 12 mmHg, subsequent long-term treatment with propranolol or nadolol plus EVL, and a second HVPG assessment after one to three months of continued NSBBs. By combining clinical data (i.e., presence of ascites or encephalopathy) plus severity of portal hypertension (HVPG ≥ 16 mmHg), they identified two groups of patients at significantly different risks of rebleeding and mortality during follow-up. “Low” risk group included patients without ascites or encephalopathy and patients with VH plus ascites or encephalopathy but HVPG < 16 mmHg. “High” risk group included patients with VH plus one among the follows: ascites or HE, HVPG ≥ 16 mmHg, and lack of response to NSBBs as defined by an HVPG decrease by at least 20% of <12 mmHg. If confirmed by prospective series, this algorithm may improve risk stratification and lead to a more tailored management of patients with cirrhosis and history of VH. In fact, as shown in previous studies for acute VH [58] and “difficult ascites” [76], anticipating a decision for TIPS may be better compared with using it as rescue therapy.

In patients receiving secondary prophylaxis for VH, assessment of baseline HVPG and its response to NSBBs may provide useful information and guide therapy [77,78,79]. In this setting, the “goal-standard” is to measure HVPG at baseline and then re-assess HVPG after chronic administration of NSBBs (i.e., after four to six weeks) [80]. However, the measurement of “acute” HVPG response to intravenous propranolol may be a preferred alternative as it would be quicker and has an acceptable correlation with chronic response [77]. In one seminal RCT by Villanueva et al., an HVPG-guided therapy based on acute response to intravenous NSBBs significantly lowered the risk of portal hypertension related complications and mortality compared with standard of care [78]. In another retrospective study including both candidates for primary and secondary prophylaxis, acute response to intravenous propranolol was independently correlated with a 50% decrease in the probability of re-bleeding (23% at 2 years vs. 46% in non-responders; *p* = 0.032) and a better survival (95% vs. 65%; *p* = 0.003) [79]. Although further evidence is required to evaluate benefits and cost of such approach, current data suggest that HVPG-guided therapy in patients who are not deemed candidates for pre-emptive TIPS, could improve the management of secondary prophylaxis by reducing costs and adverse events due to ineffective therapy [77].

### 3.2. Second Line Therapy

Current guidelines recommend covered TIPS as second line therapy of choice in patients who experience rebleeding despite combination therapy with NSBB plus EVL [5,6,7,8].

Regarding prevention of recurrent VH by TIPS, RCTs comparing uncovered TIPS vs. NSBBs plus EVL (standard of care) agreed that TIPS is very effective in preventing rebleeding, but it is associated with higher risk of over encephalopathy and does not improve survival [81]. Comparable findings were confirmed by two RCTs in which covered TIPS was used [82,83]. Therefore, TIPS is considered the treatment of choice only in patients who fail first-line therapy (NSBBs plus EVL), in whom the risk of bleeding-related mortality is very high and exceeds those associated with TIPS [5,6,7,8].

Patients who experience the first episode of VH while on primary prophylaxis with NSBBs have a higher risk of rebleeding and mortality compared to those who experience VH not being on NSBBs, despite being treated with recommended combination therapy [84]. Although the best treatment strategy in these patients is unknown, they may benefit from a more aggressive strategy, and TIPS may be considered earlier rather than later in these patients. A second group of patients in whom to consider TIPS before failure of standard therapy are those who are or who become intolerant to NSBBs. In fact, as mentioned before, NSBBs are the cornerstone of combined therapy, particularly in decompensated patients (Child B and C) [67].

In patients with cirrhosis and portal vein thrombosis who have recently bled, variceal obliteration with EVL takes longer and varices recur at a higher rate compared to patients without thrombosis [85]. Additionally, a small RCT showed that TIPS is more effective than EVL and NSBBs in preventing rebleeding in patients with cirrhosis and portal vein thrombosis, with a higher rate of thrombus resolution but without differences in mortality [61]. Patients with cirrhosis and portal vein thrombosis, which is the most common thrombotic complications in cirrhosis [86,87,88], may be a third group in which to consider TIPS earlier rather than later in the setting of secondary prophylaxis. This would be particularly important if the patient is awaiting liver transplantation, as the presence of thrombosis at time of transplantation is associated with a higher risk of post-transplant mortality [89].

A major clinical challenge in patients who receive TIPS for second-line prophylaxis of VH remains prediction of survival and prognosis (i.e., identification of patients with poor outcomes after TIPS in whom early evaluation for transplantation should be indicated). Recently, Bettinger et al. proposed to combine four simple variables (age, bilirubin, albumin and creatinine) in a new score named the “Freiburg index of post-TIPS survival” (FIPS score) [90]. In a very large cohort of patients who received TIPS for various indication, including second line prophylaxis of VH, the FIPS score was able to identify those at higher-risk for progression and death, and its prognostic discrimination was superior to other currently used score such as MELD, MELD-Na, and Child-Pugh [90].

## 4. Conclusions

Development of variceal hemorrhage in patients with cirrhosis poses a complex challenge requiring a multidisciplinary approach that is important to prevent rebleeding and improve survival. The management of variceal hemorrhage in these patients should take into consideration the severity of underlying portal hypertension and the presence (or absence) of other complications of cirrhosis, especially ascites. In patients presenting with variceal hemorrhage, the advances in the therapy of portal hypertension have resulted in lower rates of rebleeding and death, particularly for therapies associated with a decrease of portal pressure. Further improvement in risk stratification and in therapies of patients with cirrhosis and variceal hemorrhage are eagerly awaited.

## Data Availability

Not applicable.
